# Evaluating the ability of a thermal biology-informed reproduction number to explain patterns of West Nile incidence in Europe

**DOI:** 10.1016/j.onehlt.2026.101469

**Published:** 2026-06-06

**Authors:** Julian Heidecke, Hedi Katre Kriit, Peter Fransson, Jonas Wallin, Joacim Rocklöv

**Affiliations:** a Interdisciplinary Center for Scientific Computing, Heidelberg University, Germany; b Heidelberg Institute of Global Health, Heidelberg University, Germany; c Department of Epidemiology and Global Health, Umeå University, Sweden; d Department of Statistics, Lund University, Sweden

**Keywords:** West Nile virus, Incidence, Thermal biology, Transmission potential, Mathematical modelling, Vector-borne diseases, Public health

## Abstract

West Nile virus (WNV) is a growing risk to public and veterinary health in Europe, with intensifying outbreaks over recent decades coinciding with a rapidly warming climate. Thermal biology models parameterized through laboratory experiments on the mosquito vectors and the pathogen play a central role in our causal understanding of the effects of temperature on WNV transmission. We evaluated the ability of a thermal biology model of WNV's relative basic reproduction number (R_0_) to explain patterns of real-world transmission risk using monthly records of human West Nile virus neuroinvasive disease (WNND) in Europe between 2010 and 2023. We assessed spatial and temporal alignments of R_0_ estimates and WNND observations and calculated R_0_ estimates from temperature data of varying resolution, assessing the value of these estimates for WNND risk ranking compared to temperature alone. Moreover, we used generalized additive models to investigate if the effect of temperature on the WNND incidence across Europe mirrors the R_0_ temperature response, including the optimal temperature for transmission. We found that R_0_ accurately captured the seasonality of WNND, the temperatures associated with peak risk, and marginally improved WNND risk ranking compared to temperature alone. However, R_0_ poorly explained the irregular interannual incidence pattern and more limited geographical range of reported WNND cases. Additionally, nonlinear averaging of R_0_ calculations from high temporal resolution temperature data slightly improved risk ranking at elevated temperatures, whereas estimates based on average temperatures were better when lower temperatures were also included. Sensitivity analyses suggested that the validation of the optimal transmission temperature was largely informed by observations from Greece, as other countries contributed little information at such high temperatures. These findings demonstrate that thermal biology models capture important aspects of WNND risk in Europe. However, incorporation of additional ecological and epidemiological drivers is needed to develop more accurate risk predictions.

## 1 Introduction

West Nile virus (WNV) is currently the leading cause of human mosquito-borne infections in Europe [1]. WNV is maintained in a transmission cycle between birds and mosquitoes with *Culex pipiens* as the primary vector in Europe [2]. Although many *Culex* species are primarily bird-biting, they express an opportunistic feeding behavior driven by extrinsic factors, such as host availability, which results in sporadic transmission of WNV to dead-end-hosts including humans [3], [4], [5]. Among humans 20–25% of infections result in “flu-like” symptoms and about 1% develop severe neurological complications – West Nile neuroinvasive disease (WNND) – with a fatality rate of approximately 10% [6], [7]. The endemic range of WNV in Europe has been extending over the last 10–20 years partly attributed to increasingly suitable climate conditions [8], including a rapid warming with twice the temperature rise compared to the global trend [9].

Climatic factors such as temperature are affecting WNV transmission through their effects on mosquitoes, bird populations, and virus transmissibility [10]. Current evidence of the effects of temperature on avian hosts is largely based on observational studies and the key reservoir species in Europe remain poorly characterized [10], [11], [12]. In contrast, laboratory experiments have provided an understanding of the nonlinear effects of temperature on mosquito-pathogen traits, such as life-history parameters, vector competence, and the extrinsic incubation period, which can be used to parameterize temperature-driven mechanistic models of WNV transmission [13], [14]. To lower complexity, such thermal biology-informed models are often reduced to summary indices such as the basic reproduction number ($R_{0}$), which characterizes long-term average transmission assuming constant temperatures [14], [15]. $R_{0}$ describes the expected number of secondary infected hosts arising from a single infected host in a completely susceptible population, and is often used as a measure of thermal suitability for transmission [16], [17], [18], [19]. The thermal biology approach predicts a unimodal response to temperature with a mosquito- and pathogen-specific thermal optimum that optimizes the tradeoffs between the mosquito-pathogen traits [15]. A recent study estimated a thermal optimum of 24.5 °C for WNV transmission by *Culex pipiens* [14].

The lab-derived, simplified thermal biology indices and their ability to capture real-world transmission risk require validation against independent field observations [15]. In Europe and the Mediterranean basin, validations of thermal biology indices for WNV have so far been limited to comparisons with equine cases [20] or human cases and mosquito dynamics in individual countries [21], [22], [23]. The informative value of a lab-predicted optimal transmission temperature has only been investigated in the United States where a close agreement to the summer temperatures of counties with highest human WNND incidence was reported [13]. However, it is unknown if this agreement holds in Europe, where the dominant vector and host species, virus strains, and climate patterns differ compared to the United States. There have been numerous studies that described positive associations between temperature and WNV transmission risk in Europe [8], [24], [25], [26], [27], but the range of high temperatures at which WNV transmission risk begins to decline has not been documented.

Moreover, studies have applied mechanistic suitability indices at varying temporal scale, e.g., daily [23], [28] or monthly [13], [22], to assess transmission potential of WNV. The application at high temporal resolution aims to account for fluctuating temperature conditions, but it is unclear whether these indices can adequately capture such effects and whether brief periods of suitable temperatures are meaningful for transmission potential. An assessment of the informative value of calculating thermal suitability measures from high spatial and temporal resolution temperature data for describing WNV transmission potential is missing.

In this study, we evaluated the agreement between a recently developed thermal suitability model based on the relative basic reproduction number ($R_{0}^{\mathrm{rel}}$) for transmission of WNV by *Culex pipiens* [14] with reports of monthly human WNND cases in Europe between 2010 and 2023. First, we investigated the ability of $R_{0}^{\mathrm{rel}}$ to capture spatial and temporal patterns of WNND. Secondly, we compared estimates of $R_{0}^{\mathrm{rel}}$ calculated from high resolution and aggregated temperature data, evaluating which method maximizes the informative value for WNND risk ranking, and the information gained compared to temperature alone. Thirdly, using statistical models we investigated if the unimodal temperature response and optimum temperature of transmission predicted by $R_{0}^{\mathrm{rel}}$ are reflected in the WNND incidence in Europe, and quantified the response of WNND to $R_{0}^{\mathrm{rel}}$.

## 2 Methods

### 2.1 Data

#### 2.1.1 Temperature

We obtained daily two meter air temperature data at 0.1° resolution for the time period 2010–2023 from the ERA5-Land dataset [29], [30]. To match the spatial resolution of the outcome data, we aggregated the temperature data to NUTS3 regions in two ways: an unweighted spatial average and a population-weighted spatial average based on the Eurostat 2021 population census providing a 1km^2^ grid [31]. To consider lagged effects, we calculated 1/2/3- and 4-month moving average values of temperature for each month in our study period, including the values of the focal and prior months. Throughout the main analysis, we use the population-weighted 3-month moving average values of temperature as the monthly predictor.

#### 2.1.2 Temperature-dependent $R_{0}^{\mathrm{rel}}$ model

We used a model of the relative basic reproduction number $\left(R_{0}^{\mathrm{rel}}\right)$ for *Culex pipiens* based on a previous study published by the authors [14]. This relative version isolates the nonlinear impact of temperature on $R_{0}$ via several mosquito-pathogen traits. In the previous study, Bayesian hierarchical models that account for variability between mosquito species and experiments were applied to lab-based mosquito-pathogen trait data to fit temperature response functions for each trait. We updated the previously developed model [14] through a sampling scheme that ensures that traits representing survival probabilities stay between 0 and 1, without the need for an upper truncation. This update had a very small impact on the thermal optimum and no impact on the mean thermal limit estimates for *Culex pipiens* where $R_{0}^{\mathrm{rel}}$ becomes zero. For a full description and the model result comparison, please see Supplementary Section B. We used the updated median estimates for $R_{0}^{\mathrm{rel}}$ with respect to temperature scaled to the interval [0,1] to calculate thermal suitability estimates from the temperature data.

#### 2.1.3 WNV cases

Anonymized case-based human WNV data reported by European Union/European Economic Area (EU/EEA) member states for 2010–2023 was obtained from The European Surveillance System (TESSy) of the European Center for Disease Prevention and Control (ECDC). We included cases of WNND with a known place of infection and aggregated the data to monthly number of cases for each NUTS3 region. We excluded asymptomatic and unspecific clinical manifestation cases to minimize surveillance biases. For the calculation of incidence rates we used annual population data from Eurostat [32].

### 2.2 Spatial-temporal patterns of $R_{0}^{\mathrm{rel}}$ and WNND

We explored the alignment between spatial and temporal patterns of WNND and $R_{0}^{\mathrm{rel}}$. For the spatial comparison, we visually contrasted a map of the total incidence of WNND of each region with a map of 3-month moving average $R_{0}^{\mathrm{rel}}$ during July–September averaged over years. For the temporal comparison, we focused on hotspot regions in Hungary, Romania, Italy, and Greece, which we defined as the largest contiguous groups of NUTS3 regions with at least one case in at least six years. We visually compared time series of $R_{0}^{\mathrm{rel}}$ with monthly case counts standardized by region to assess overlaps in seasonality and year to year variability.

These comparisons showed that many regions with similar temperature conditions differed in whether they reported WNND cases or not and that even regions with a history of WNND frequently experienced years with no reported cases. Therefore, we considered these observations unlikely to provide informative signal on the relationship between temperature and WNV transmission intensity and removed years with no cases in a given NUTS3 region for all subsequent analyses.

### 2.3 Ranking WNND risk: temperature vs. $R_{0}^{\mathrm{rel}}$ and impact of aggregation method

**Fig. 1:**
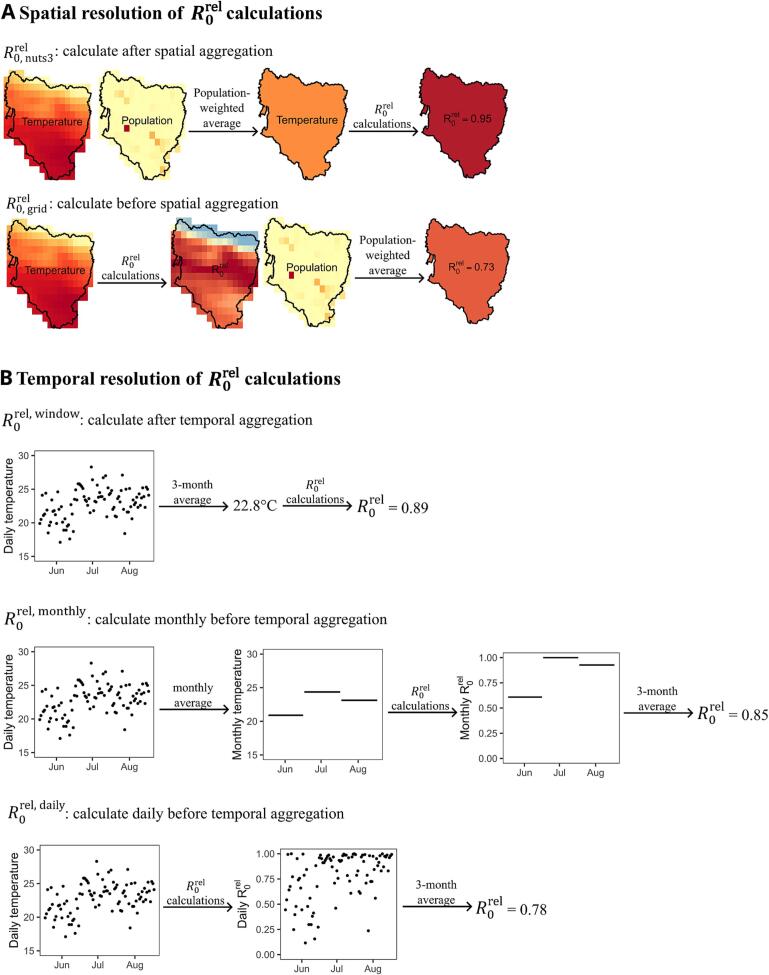
Illustration of calculation methods of 3-month moving average values of $R_{0}^{\mathrm{rel}}$ from temperature data before or after spatial (A) and temporal (B) aggregation.

We compared the informative value of temperature and $R_{0}^{\mathrm{rel}}$ for ranking WNND risk. However, there is uncertainty whether $R_{0}^{\mathrm{rel}}$ calculations should occur before or after spatial and temporal data aggregation, because the derivation of $R_{0}^{\mathrm{rel}}$ assumes large, well-mixed populations under constant temperatures, which conflicts with real-world conditions. Therefore, to identify the method with highest information value, we computed $R_{0}^{\mathrm{rel}}$: (i) directly from NUTS3 3-month moving average temperature and (ii) prior to temporal (daily and monthly) and spatial (grid-level) aggregation (Fig. 1). We quantified rank correlations of the $R_{0}^{\mathrm{rel}}$ estimates retrieved from the different aggregation methods and of temperature in relation to the WNND incidence. We calculated Kendall's $\tau_{B}$, a non-parametric measure adjusted for ties, with higher values indicating a stronger monotonic association between two variables [33]. The statistical significance of differences between two Kendall's $\tau_{B}$ was tested with two-sided permutation tests under the null hypothesis of equality. For details, please see Supplementary Section A.

Evidence from a lab-based study on Malaria mosquitoes suggests that calculating thermal suitability after temporal temperature aggregation could provide more accurate predictions near thermal limits, while the opposite could be true for predicting suitability around the temperature optimum [34]. To assess whether the same pattern is observed in the ranking performance of the different $R_{0}^{\mathrm{rel}}$ calculation methods, we first calculated rank correlations using the full dataset and, secondly, repeated the analysis after excluding observations with 3-month moving average temperature below 15 °C to remove observations around the lower thermal limit where the likelihood of transmission is small. All other analyses used the calculation method for $R_{0}^{\mathrm{rel}}$ that performed best in the full dataset.

### 2.4 Comparing the temperature effect on WNND incidence with $R_{0}^{\mathrm{rel}}$

First, we used generalized additive models (GAMs) to estimate the empirical temperature-WNND relationship, assessing whether it exhibits a unimodal form and peak temperature consistent with the response predicted by lab-based $R_{0}^{\mathrm{rel}}$ [35]. Case counts were modeled using a negative binomial distribution with a log-link function to account for overdispersion, with log(population) included as an offset. For temperature effects we used penalized cubic regression splines, including a shared smooth term (basis dimension k = 20) to capture the overall relationship across countries and a country-specific smooth term (k = 5) to allow deviations from the shared pattern. The country-specific term was specified as a constrained factor-smooth interaction with sum to zero constraints. Smoothness was estimated by restricted maximum likelihood with an additional shrinkage penalty. We tested if population-weighted 3-month moving average temperatures were the best performing variable by fitting separate GAMs for the unweighted and population-weighted 1/2/3/4-month moving averages and compared models using AIC (Table S1 in Supplementary). For computational efficiency, this initial selection was performed without the country-specific term. We focused on GAMs incorporating a single time window to maintain comparability with the $R_{0}^{\mathrm{rel}}$ temperature response which collapses mosquito population and infection processes into a single metric. Unlike more flexible statistical models [27] or dynamic simulations [28], $R_{0}^{\mathrm{rel}}$ is not designed to resolve stratified or temporally ordered effects of temperature on transmission.

Second, we followed the same GAM approach for $R_{0}^{\mathrm{rel}}$ with basis dimension k = 10 for the shared smooth term and k = 3 for the country-specific smooth term. We evaluated if the resulting AIC value was in a similar range as for the temperature GAM, despite the nonlinear constraints imposed by $R_{0}^{\mathrm{rel}}$. We also visually assessed the $R_{0}^{\mathrm{rel}}$-WNND relationship for remaining nonlinearities.

Third, we tested the robustness of the derived relationships to the inclusion of climatic, land use, and socio-economic adjustment variables. We extracted the percentage of urban fabric and arable land in each region from the CORINE Land Cover database [36], the population-weighted 3-month moving cumulative precipitation from ERA5-Land [29], as well as the yearly gross domestic product per capita and the percentage of population aged over 65 from Eurostat [32]. These were incorporated into the GAMs as linear effects. Adjusted relationships were calculated as the conditional response of incidence to temperature/$R_{0}^{\mathrm{rel}}$, keeping the adjustment variables at their median.

### 2.5 Sensitivity analysis

Observations with a monthly WNND incidence above 5.3 cases per 100,000 (top 1.9% of all positive incidence observations) were confined to regions in Greece, one of the few countries in Europe that implements an active rather than a passive WNV surveillance system on people [37]. Moreover, Greece accounted for about 59% of the temperature observations with 3-month moving averages exceeding the lab-based optimum of 24.4 °C. To assess the sensitivity of our results to the influence of Greece, we repeated our analysis after excluding Greece from the dataset.

All analyses were conducted in R v4.4.2 [38].

## 3 Results

### 3.1 Summary of data

**Fig. 2:**
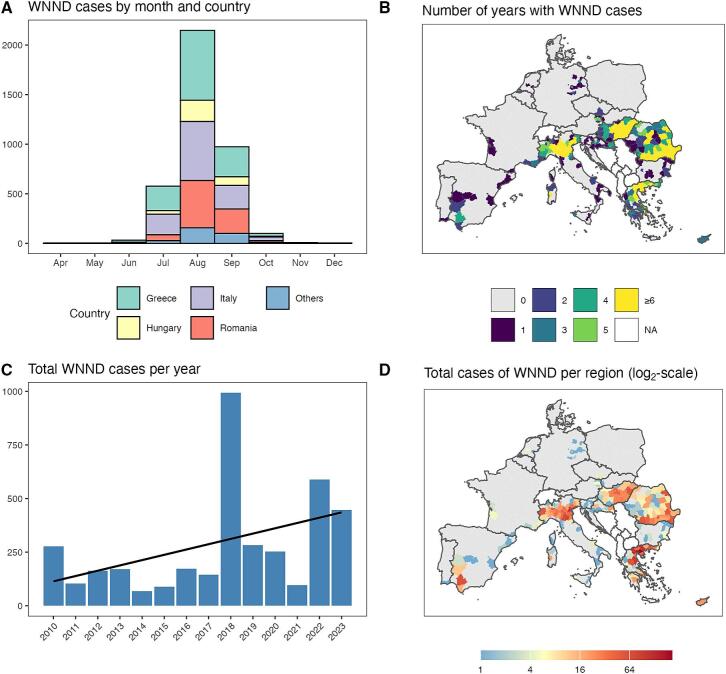
WNND cases between 2010 and 2023. (A) The number of cases stratified by country and month. Countries with less than 100 cases were grouped into “Others”. (B) The number of years with at least one case per NUTS3 region. (C) The total number of WNND cases per year with trend line. (D) The total number of WNND cases per NUTS3 region. Grey areas on the maps correspond to NUTS3 regions that did not report any WNND case. To improve visual focus, we excluded from the maps several northern EU/EEA countries that did not report any case. Blank areas correspond to non-EU/EEA regions.

A total of 3844 human WNND cases were reported during the study period. Most cases (>96%) were reported from July to September (Fig. 2A). Over 92% of cases originated from Greece, Italy, Romania, and Hungary (Fig. 2A). Within these countries, the highest and most persistent case occurrence was observed in northern Greece and Italy, southern Romania, and eastern Hungary where several regions reported cases in at least six years (Fig. 2B and D). We observed an expansion of the spatial distribution and rising trend in the total number of cases reported per year overlaid by strong interannual variation (Fig. 2C) over the study period. Northern regions in Germany and the Netherlands (Fig. 2B) reported their first WNND cases in 2019 and 2020, respectively.

The mean population-weighted 3-month moving average temperature during July to September in regions with at least one case in a given year was 22.3 °C (range: 14.1–28.7 °C), with lowest average in the Netherlands (17.5 °C; range: 15.9–18.7 °C) and highest in Cyprus (27.6 °C; range: 26.1–28.6 °C). Only 17.5% of these temperatures across all countries exceeded the lab-based optimal temperature of 24.4 °C.

### 3.2 Spatial-temporal comparison

**Fig. 3:**
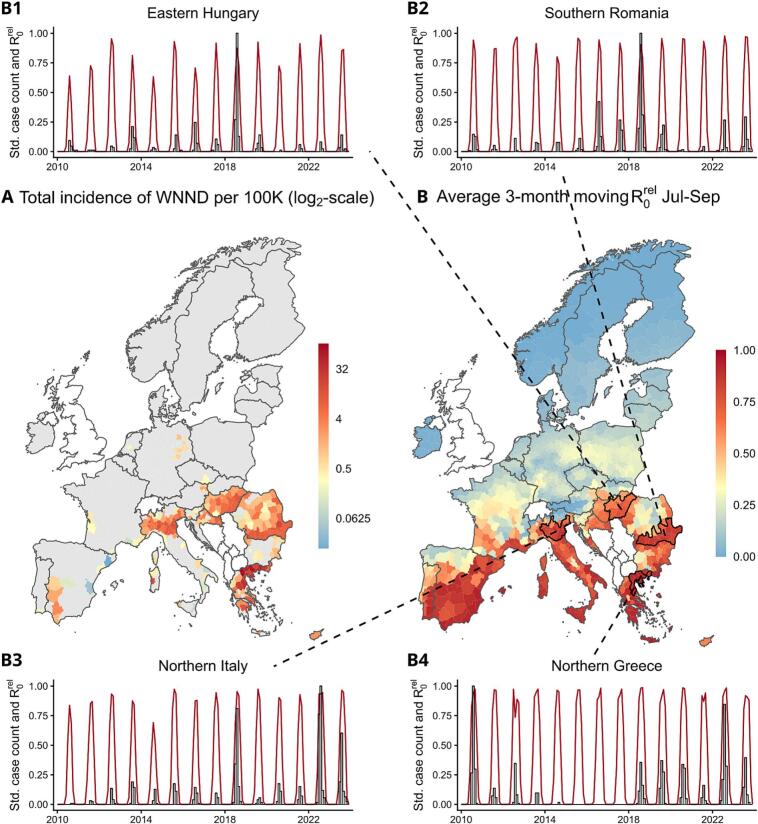
Maps of the total incidence of WNND in 2010–2023 per NUTS3 region (A) and mean 3-month moving average $R_{0}^{\mathrm{rel}}$ in July–September averaged over years (B). Blank areas correspond to non-EU/EEA regions. The time series (B1–4) present standardized monthly WNND case counts (grey bars) and 3-month moving average of $R_{0}^{\mathrm{rel}}$ (red lines) for four current hotspot areas in eastern Hungary (B1), southern Romania (B2), northern Italy (B3), and northern Greece (B4). (For interpretation of the references to colour in this figure legend, the reader is referred to the web version of this article.)

Figs. 3A-B show a spatial alignment between the distribution of the total incidence of WNND across years with the mean 3-month moving average $R_{0}^{\mathrm{rel}}$ from July–September. However, comparable values of $R_{0}^{\mathrm{rel}}$ occurred in regions with and without reported cases, suggesting that temperatures conducive for transmission indicated by $R_{0}^{\mathrm{rel}}$ extend beyond the observed distribution of cases (Fig. 3A-B). Temporal patterns of WNND cases and 3-month moving average $R_{0}^{\mathrm{rel}}$ in four WNV hotspot regions suggested agreements in seasonality (time window and peak of transmission), but that $R_{0}^{\mathrm{rel}}\;$ offers limited information about the interannual variability of WNND (Fig. 3B1—B4).

### 3.3 Rank correlation and aggregation methods

$R_{0}^{\mathrm{rel}}$ provided only a small statistically significant improvement of the informative value for ranking the WNND incidence compared to temperature ($\tau_{B}$=0.440 vs. $\tau_{B}$=0.419) (Table 1). On the unrestricted dataset, calculations of $R_{0}^{\mathrm{rel}}$ after spatial and temporal aggregation resulted in the highest rank correlation (Table 1). Contrasting with this finding, after removing observations with 3-month moving average temperature values below 15 °C, calculations of $R_{0}^{\mathrm{rel}}$ at finer temporal resolution before aggregation improved the rank correlation. In the restricted dataset, however, the difference of rank correlations of the best performing $R_{0}^{\mathrm{rel}}$ calculation method ($\tau_{B}$=0.413) and temperature ($\tau_{B}$=0.408) was not statistically significant anymore. Calculating $R_{0}^{\mathrm{rel}}$ at the native spatial resolution of the temperature data before spatial aggregation consistently weakened the rank correlation with the monthly WNND incidence (Table 1).

**Table 1:** Rank correlation coefficients of 3-month moving average temperature and $R_{0}^{\mathrm{rel}}$ with the monthly WNND incidence. Correlation coefficients within the same column that share the same superscript letter are not significantly (p>0.05) different from one another.

Predictor	Kendall's $\tau_{B}$ with monthly WNND incidence	
	No restriction	Limited to temperatures >15 °C
Temperature	0.419^ab^	0.408^abc^
$\;R_{0,\text{nuts}3}^{\mathrm{rel},\text{daily}}$	0.420^b^	**0.413**^**c**^
$R_{0,\text{nuts}3}^{\mathrm{rel},\text{monthly}}$	0.426^e^	0.409^acf^
$R_{0,\text{nuts}3}^{\mathrm{rel},\text{window}}$	**0.440**^**f**^	0.401^df^
$R_{0,\text{grid}}^{\mathrm{rel},\text{daily}}$	0.419^a^	0.411^a^
$R_{0,\text{grid}}^{\mathrm{rel},\text{monthly}}$	0.423^c^	0.405^bd^
$R_{0,\text{grid}}^{\mathrm{rel},\text{window}}$	0.431^d^	0.393^e^

### 3.4 GAM analysis

**Fig. 4:**
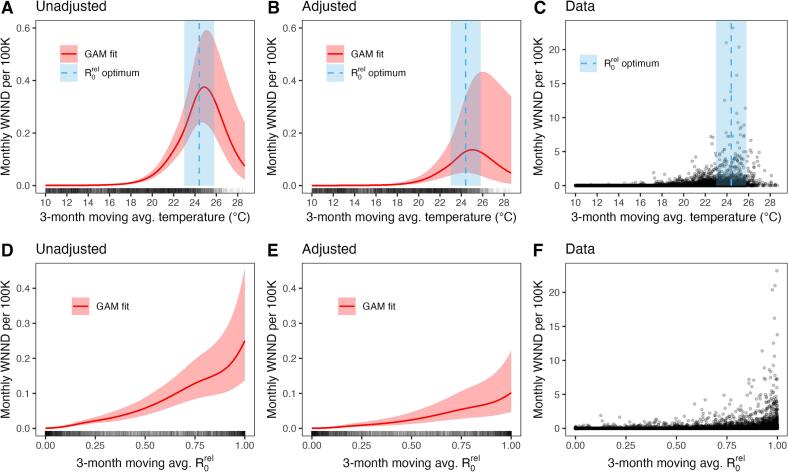
The shared response of the monthly WNND incidence to 3-month moving average temperature (A, B) and $R_{0}^{\mathrm{rel}}$ (D, E) without (A, D) and with (B, E) adjustment to additional variables. GAM mean predictions with approximate 95% CI (red solid line and ribbon) and mean and 95% CI of the optimal temperature of $R_{0}^{\mathrm{rel}}$ (blue dashed line and ribbon). Rug plots on top of x-axes show the distribution of 3-month moving average temperature/$R_{0}^{\mathrm{rel}}$. (C), (F) Corresponding raw data. (For interpretation of the references to colour in this figure legend, the reader is referred to the web version of this article.)

**Fig. 5:**
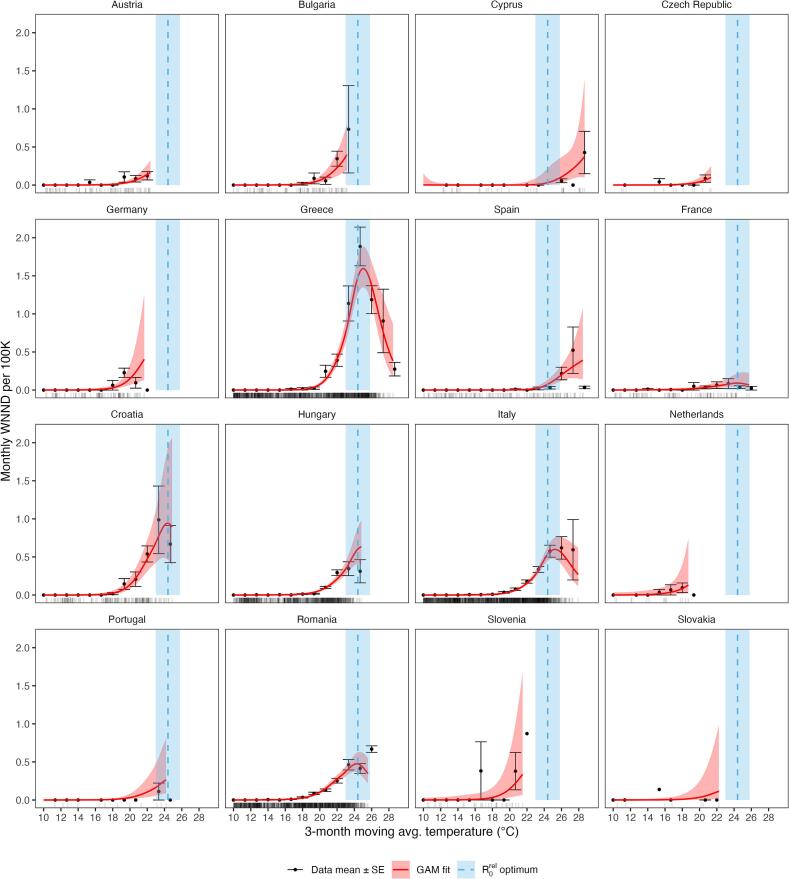
The response of the monthly WNND incidence to 3-month moving average temperature by country. Binned data means ± standard errors for equally distant bins (black dots and bars), GAM mean predictions with approximate 95% CI (red solid lines and ribbons) and mean and 95% CI of the optimal temperature of $R_{0}^{\mathrm{rel}}$ (blue dashed line and ribbon). The rug plots on top of the x-axes show the distribution of 3-month moving average temperature. (For interpretation of the references to colour in this figure legend, the reader is referred to the web version of this article.)

We observed a unimodal response of the monthly WNND incidence to temperature (Fig. 4A), with an optimum temperature estimated at 24.9 °C (95% CI: 24.5–25.4 °C), which is close to the lab-based optimum of $R_{0}^{\mathrm{rel}}$ at 24.4 °C (95% CI: 22.9–25.9 °C). Across countries, the unimodal temperature response was most clearly supported in Greece with optimal temperature estimated at 25.1 °C (95% CI: 24.7–25.4 °C), whereas other countries had a limited number of observations at high temperatures (Fig. 5). We noted a substantial variability in the monthly WNND incidence around the optimum temperature that remained unexplained (Fig. 4C). The association between $R_{0}^{\mathrm{rel}}$ and the WNND incidence was monotonic but non-linear, with an accelerating increase in incidence at higher $R_{0}^{\mathrm{rel}}$ (Fig. 4D and S4 in Supplementary).

After adjusting the GAMs for additional climatic, land cover, and socio-economic variables the estimated optimal temperature increased to 25.1 °C (95% CI: 24.5–26.1 °C) (Fig. 4B) and in Greece to 25.9 °C (95% CI: 25.2–27.0 °C) (Fig. S3 in Supplementary). Additionally, the uncertainty in the temperature-WNND relationship increased at temperatures around and above the optimum (Fig. 4B). The relationship between $R_{0}^{\mathrm{rel}}$ and WNND incidence remained qualitatively unchanged after adjustment (Fig. 4E and Fig. S5 in Supplementary). For both the adjusted and unadjusted, the GAM using $R_{0}^{\mathrm{rel}}$ as predictor had a slightly higher AIC than the temperature GAM (Table S1 in Supplementary).

### 3.5 Sensitivity analysis

After removing observations from Greece, we observed a high uncertainty in distinguishing the temperature-WNND relationship as unimodal or monotonic (Fig. S6A-C in Supplementary). The relationship between $R_{0}^{\mathrm{rel}}$ and WNND remained monotonic and nonlinear (Fig. S6D-F in Supplementary) and $R_{0}^{\mathrm{rel}}$ continued improving rank correlation with the WNND incidence compared to temperature (Table S5 in Supplementary). We noted a change in the optimal calculation method for $R_{0}^{\mathrm{rel}}$ on the dataset restricted to 3-month moving temperatures above 15 °C: unlike in the dataset with observation from Greece, calculating $R_{0}^{\mathrm{rel}}$ at the native spatial resolution of the temperature data before spatial aggregation slightly improved rank correlation with the monthly WNND incidence (Table S5 in Supplementary).

## 4 Discussion

Mechanistic models informed by thermal biology provide tools for estimating thermal suitability for the transmission of vector-borne diseases. In this study, we assessed the ability of a recently published lab-based thermal suitability model, $R_{0}^{\mathrm{rel}}$ [14], to describe WNV transmission risk in Europe. We demonstrate that $R_{0}^{\mathrm{rel}}$ slightly improves WNND risk ranking relative to temperature alone and found a close alignment between the lab-predicted optimal transmission temperature and the temperature of peak WNND risk. By conducting this evaluation against human WNND data at a monthly scale across NUTS3 regions, our analysis extends previous literature on thermal biology-driven suitability predictions that were limited to individual countries [21], [22], [23] or used equine cases [20] in Europe and the Mediterranean basin. Moreover, we compared alternative calculation methods for $R_{0}^{\mathrm{rel}}$, investigating the application of thermal biology models to observational data and potential benefits of high-resolution temperature data as their input.

The 3-month moving average $R_{0}^{\mathrm{rel}}$ aligned well with the observed seasonality of human WNND cases, with a general temporal overlap and agreement in peak timing. In contrast to a previous study that reported a 2-month lag between monthly thermal suitability and observed cases, the use of a 3-month moving average removed this temporal offset [13]. However, $R_{0}^{\mathrm{rel}}$ did not fully explain the interannual variability and the more limited geographical range of reported WNND cases. These discrepancies underline limitations of $R_{0}^{\mathrm{rel}}$ which currently does not consider host immunity dynamics, climatic conditions beyond the effect of temperature on mosquito-pathogen traits, or land use which could explain differences in year-to-year WNV variability [25], [39], [40], or the limited geographical range of human cases [8], [24], [25], [41], [42]. The value of $R_{0}^{\mathrm{rel}}$ as a risk indicator would likely increase, if the mechanistic model is extended to consider some of these factors. Additionally, parts of the greater suitable area highlighted by $R_{0}^{\mathrm{rel}}$ could be a result of underreporting or may reflect that human cases are not always a reliable proxy for WNV amplification intensity [43], [44], [45].

Using the full dataset, the 3-month moving average $R_{0}^{\mathrm{rel}}$ slightly improved rank correlation with monthly WNND incidence over temperature, with best performance obtained when $R_{0}^{\mathrm{rel}}$ was calculated after temporal aggregation of the temperature data. After excluding observations below 15 °C, calculation of $R_{0}^{\mathrm{rel}}$ before temporal aggregation performed best but was no longer significantly better than temperature, suggesting that the thermal optimum provided limited information for ranking WNND incidence. Nonetheless, the observed pattern, that nonlinear averaging of $R_{0}^{\mathrm{rel}}$ calculations at high temporal resolution might be preferred to estimate thermal suitability at temperatures around the thermal optimum, whereas average temperature inputs may be more informative near thermal limits, is in line with evidence from a laboratory study on Malaria mosquitoes [34]. Calculating $R_{0}^{\mathrm{rel}}$ on the temperature grid before spatial aggregation to NUTS3 regions resulted in slightly reduced rank correlation, which was contra intuitive, since one might assume that more granular spatial data would increase the informative value of $R_{0}^{\mathrm{rel}}$. After removing data from Greece, $R_{0}^{\mathrm{rel}}\;$ calculations before spatial averaging indeed improved rank correlation. Greece has a long, complex coastline and it has been shown that ERA5-Land temperature accuracy decreases with proximity to the coast in Southeast China [46], which might explain the reduction of the informative value of $R_{0}^{\mathrm{rel}}$ calculations at the grid cell-level. Therefore, the value of calculating $R_{0}^{\mathrm{rel}}$ at high spatial resolution might depend on the quality of temperature data.

Given the small differences observed in rank correlation across $R_{0}^{\mathrm{rel}}$ calculation methods, these results need to be interpreted with caution and further research is needed to provide definite guidance. Further development of thermal suitability models that can inherently account for fluctuating conditions would be highly valuable [34], [47], [48].

The temperature-WNND relationship estimated with GAMs was unimodal with similar optimum temperature as predicted by the lab-based $R_{0}^{\mathrm{rel}}$, which has previously been demonstrated in a study on human WNND at county-level in the United States [13]. The similarity between laboratory, United States, and European optimal temperature estimates suggests a notable consistency in the temperature sensitivity of WNV transmission across transmission settings. Nevertheless, we observed a small difference between the lab-based and real-world estimated optimal transmission temperature together with a decreased GAM performance when using $R_{0}^{\mathrm{rel}}$ as predictor instead of temperature. These differences might be due to an oversimplification in lab-environments or may reflect temperature-dependent processes that are not incorporated in $R_{0}^{\mathrm{rel}}$. Alternatively, the difference might be the result of using human cases as the outcome, which could result in increased temperature estimates compared to the bird-mosquito amplification cycle. The adjusted GAMs indicated a higher optimal temperature, suggesting that other factors may partly explain the decline of WNND at high temperatures. However, uncertainty in the association around an above the optimal temperature increased, suggesting that the low number of observations with prolonged high temperature conditions together with the crude spatial scale of our study prevented to accurately separate the effects of temperature and of the adjustment variables.

In contrast to the unimodal association between temperature and WNND identified in this study, several analyses in European and Mediterranean settings have reported strictly positive associations between temperature and WNV infections in humans [8], [24], [25], [26], [27]. This difference could result from analytical choices, such as using binary instead of incidence data as the outcome variable [8], [24], higher aggregation of the outcome variable [8], [24], [25], incorporating a longer list of predictors [8], [24], [25], or a focus on short-term effects and a more conservative approach to model flexibility [27]. Currently, temperatures in Europe rarely exceed the optimal range for WNV transmission for prolonged periods, making it difficult to detect the decline in transmission risk at extreme high temperatures. However, the frequency of high temperatures is expected to increase in the future due to climate change. Therefore, accounting for unimodal temperature effects on transmission risk in Europe will become increasingly important, especially in projections studies [41], [49], [50].

### 4.1 Limitations

Although restricting to cases of WNND, the data might be subject to biases resulting from differences in diagnostic approaches, surveillance efforts, and control measures applied across Europe [37]. We allowed country-specific deviations in our GAM analysis but otherwise observations were pooled. Therefore, additional spatial or temporal heterogeneity is not accounted for. Our sensitivity analysis suggested that the unimodal temperature response and peak WNND temperature estimate is strongly driven by observations from Greece. The limited number of observations at temperatures above the lab-predicted optimal temperature in other countries prevented to robustly investigate a potential heterogeneity in the temperature response across Europe. In addition, while $R_{0}^{\mathrm{rel}}$ is derived from a mechanistic model, our statistical analysis is correlative and does not imply causality. Future studies on the effects of temperature and other factors on WNV may benefit from principles of causal inference to strengthen the derived relationships [51]. Moreover, we only utilized human infection data. To develop a deeper understanding of the ability of thermal biology-driven models to describe WNV transmission suitability in Europe a similar methodology could be applied to mosquito and animal infections, if appropriate data is available.

## 5 Conclusion

In conclusion, this study demonstrates that a lab-derived thermal biology-driven model improves risk ranking of WNND in Europe compared to temperature alone, and captures features of observed dynamics, including the seasonality and approximating the temperature of peak WNND risk. However, substantial variability in WNND remains unexplained by thermal suitability alone. This supports the use of thermal biology-driven models as mechanistic indicators of temperature-related changes in WNV transmission potential, while highlighting the need to integrate or extend these models with additional drivers and address their simplifying assumptions for more comprehensive risk assessments.

## Declaration of AI and AI-assisted technologies in the manuscript preparation process

During the preparation of this work the authors used ChatGPT (GPT-5) to improve readability, clarity, and language of the manuscript. After using this tool/service, the authors reviewed and edited the content as needed and take full responsibility for the content of the published article. ChatGPT was also used to assist with computer code development and debugging. JH reviewed and edited the suggestions as needed and remains fully accountable for the final code and output.

## CRediT authorship contribution statement

**Julian Heidecke:** Writing – review & editing, Writing – original draft, Visualization, Validation, Software, Methodology, Investigation, Funding acquisition, Formal analysis, Data curation, Conceptualization. **Hedi Katre Kriit:** Writing – review & editing, Visualization, Validation, Supervision. **Peter Fransson:** Writing – review & editing, Validation, Supervision, Methodology, Investigation. **Jonas Wallin:** Writing – review & editing, Validation, Methodology, Investigation. **Joacim Rocklöv:** Writing – review & editing, Validation, Investigation, Conceptualization.

## Funding

JR received support from the 10.13039/100005156Alexander von Humboldt Foundation through the funding instrument of an Alexander von Humboldt Professorship endowed by the 10.13039/501100002347Federal Ministry of Education and Research in Germany. The study was supported by funding from the IDAlert project (http://idalertproject.eu) funded by the European Union’s Horizon Europe programme (Grant agreement 101057554 to JR) and by funding from the 10.13039/100009139German Center for Infection Research (DZIF) (grant number TTU 01.823 to JR and JH). The funders had no role in study design, data collection and analysis, decision to publish, or preparation of the manuscript.

## Declaration of competing interest

Joacim Rockloev reports financial support was provided by German Center for Infection Research. Julian Heidecke reports financial support was provided by German Center for Infection Research. Joacim Rockloev reports financial support was provided by Alexander von Humboldt Foundation. Joacim Rockloev reports financial support was provided by Horizon Europe. If there are other authors, they declare that they have no known competing financial interests or personal relationships that could have appeared to influence the work reported in this paper.

## Data Availability

Human WNV cases data were made available upon request of the authors from The European Surveillance System (https://www.ecdc.europa.eu/en/publications-data/european-surveillance-system-tessy). Meteorological data are openly available at the Copernicus Climate Data Store (https://cds.climate.copernicus.eu). Population and socioeconomic data are openly available at Eurostat (https://ec.europa.eu/eurostat/de/). Land cover data is available from the CORINE Land Cover database (https://land.copernicus.eu/en/products/corine-land-cover). The R code underlying our analyses is accessible at: https://github.com/julehe/WNV-R0-vs-cases
