# Supplementary material for: Evaluating the ability of a thermal biology-informed reproduction number to explain patterns of West Nile incidence in Europe
Source: One Health. 2026 Jun 6;22:101469. doi: 10.1016/j.onehlt.2026.101469 (PMC13262279; doi:10.1016/j.onehlt.2026.101469)
Supplement: Supplementary file 1 — Supplementary material [file mmc1.pdf]

Supplementary information to "Evaluating the ability of a thermal biology-informed reproduction number to explain patterns of West Nile incidence in Europe"

Julian Heidecke[a,b], Hedi Katre Kriit[b,c], Peter Fransson[a], Jonas Wallin[d], Joacim Rocklöv[a,b,c*]

[a]Interdisciplinary Center for Scientific Computing, Heidelberg University, Germany

[b]Heidelberg Institute of Global Health, Heidelberg University, Germany

[c]Department of Epidemiology and Global Health, Umeå University, Sweden

[d]Department of statistics, Lund University, Sweden

*Corresponding author

E-mail: joacim.rocklov@umu.se

Phone number: +4915257908947

A DETAILS ON PERMUTATION TEST AND SUMMARY OF GAM RESULTS 2

B DETAILS ON THERMAL BIOLOGY MODEL AND UPDATES TO TEMPERATURE RESPONSE ESTIMATES 2

C ADDITIONAL COUNTRY-LEVEL AND SENSITIVITY RESULTS 9

REFERENCES 13

A Details on permutation test and summary of GAM results

For testing the statistical significance of differences between two Kendall's τ_B, we performed two-sided permutation tests under the null hypothesis of equality. Specifically, when testing the difference of the Kendall's τ_B of two variables A and B with the WNND incidence, we conducted 10000 permutations of the ranks of A with the ranks of B and derived a p-value by calculating the percentage of permutations that would lead to a more extreme difference in Kendall's τ_B coefficients. We considered p-values smaller than 0.05 as indicating statistical significance.

Table S1. Summary of GAM results.

Predictors	Estimated degrees of freedom	Deviance explained	AIC
3-month moving average with country effects			
Population-weighted temperature	29.65	71.2%	8921.26
Population-weighted temperature and adjustments	34.06	72.9%	8710.47
Population-weighted $R_{0,\text{nuts3}}^{\text{rel, window}}$	29.48	70.4%	9010.69
Population-weighted $R_{0,\text{nuts3}}^{\text{rel, window}}$ and adjustments	33.81	71.7%	8855.36
3-month moving average without country effects			
Temperature	7.76	65.9%	9440.71
Population-weighted temperature	8.17	68.3%	9191.82
$R_{0,\text{nuts3}}^{\text{rel, window}}$	7.64	65.8%	9450.21
Population-weighted $R_{0,\text{nuts3}}^{\text{rel, window}}$	6.72	67.5%	9291.90
4-month moving average without country effects			
Temperature	8.65	61.4%	9873.29
Population-weighted temperature	7.82	63.9%	9640.13
$R_{0,\text{nuts3}}^{\text{rel, window}}$	7.28	60.8%	9911.87
Population-weighted $R_{0,\text{nuts3}}^{\text{rel, window}}$	7.64	63.8%	9648.49
2-month moving average without country effects			
Temperature	7.35	63.3%	9683.27
Population-weighted temperature	7.07	65.0%	9520.02
$R_{0,\text{nuts3}}^{\text{rel, window}}$	7.92	62.0%	9808.26
Population-weighted $R_{0,\text{nuts3}}^{\text{rel, window}}$	7.66	60.9%	9913.42
1-month moving average without country effects			
Temperature	10.27	53.5%	10507.69
Population-weighted temperature	9.32	54.5%	10429.44
$R_{0,\text{nuts3}}^{\text{rel, window}}$	8.81	50.9%	10691.10
Population-weighted $R_{0,\text{nuts3}}^{\text{rel, window}}$	8.84	50.0%	10758.19

B Details on thermal biology model and updates to temperature response estimates

Based on the authors previous work [1], we used a relative version of the basic reproduction number (R_0^{rel}) as a measure of WNV thermal suitability (Eq. S1).

$$R_0^{\text{rel}}(T) = \frac{M^*(T)a(T)^2 b_M(T)e^{-\mu_M(T)\text{EIP}(T)}}{\mu_M(T)} \qquad (S1)$$

This relative version isolates the effect of temperature (T) on R_0 through the non-linear interaction of several mosquito-pathogen traits. The traits include: the adult mosquito biting rate $a(T)$, mosquito infection probability $b_M(T)$, adult mosquito mortality rate $\mu_M(T)$, and the length of the extrinsic incubation period EIP(T). Effects of temperature on the mosquito abundance $M^*(T)$ are incorporated through an equilibrium expression (Eq. S2) derived from a stage-structured mosquito population dynamics model [1].

$$M^*(T) = \begin{cases} \dfrac{\omega^2 \beta(T) p_E(T) \delta_J(T)^2}{\mu_M(T)^2}\left[1 - \dfrac{\mu_M(T)}{\omega\beta(T)p_{EJ}(T)}\right], & \dfrac{\omega\beta(T)p_{EJ}(T)}{\mu_M(T)} \leq 1 \\ 0, & \text{else} \end{cases} \qquad (S2)$$

The equilibrium expression includes additional temperature dependent traits independent of the pathogen: the egg laying rate $\beta(T)$, egg viability $p_E(T)$, juvenile development rate $\delta_J(T)$, and the egg to adult survival probability $p_{EJ}(T)$ which is the product of $p_E(T)$ with the juvenile survival probability $p_J(T)$. We set the proportion of female mosquitoes at adult emergence ω to 0.5.

In the previous study, Bayesian hierarchical models that account for variability between mosquito species and experiments were applied to lab-based mosquito trait data measured under constant temperatures to fit temperature response functions for each trait [1]. In the present study, we changed the estimation of mosquito life history-traits that describe probabilities, namely the egg viability p_E, and the juvenile survival probability p_J. The updated sampling ensures that these traits stay below 1 without the need for an upper truncation. In the following, we denote the standard cumulative gaussian distribution by Φ, the normal distribution by N, the half-normal distribution by HN, and the gamma distribution by Γ.

We model p_E and p_J by a modified quadratic function:

$$f^Q(T; q, T_{\min}, T_{\max}) = \begin{cases} q(T - T_{\min})(T_{\max} - T), & T_{\min} < T < T_{\max} \\ 0, & \text{else} \end{cases}$$

Instead of sampling q in the MCMC scheme as described in our previous work [1], we sample a parameter χ which we transform into the value f_{opt} that f^Q reaches at the optimal temperature $T_{\text{opt}} = (T_{\max} + T_{\min})/2$ by applying a standard cumulative Gaussian distribution:

$$f_{\text{opt}} = \Phi(\chi; 0,1)$$

The parameter q is then obtained through the relationship:

$$f_{\text{opt}} = f^Q\left(T_{\text{opt}}; q, T_{\min}, T_{\max}\right) = q\left(\frac{T_{\max} - T_{\min}}{2}\right)^2$$

The image of the cumulative Gaussian distribution ensures that $f_{\text{opt}} \in (0,1)$. For p_J, we used the following hyperpriors for the population-level mean and the between-species and between-experiment standard deviations of $\chi, T_{\min}, T_{\max}$:

$$\mu_\chi \sim N(0.5,2)$$
$$\sigma_\chi \sim N(0,1)$$
$$\sigma_\chi^{\text{exp}} \sim N(0,1)$$
$$\mu_{T_{\min}} \sim N(10,10^2)$$
$$\sigma_{T_{\min}} \sim HN(0,10^2)$$

$$\sigma_{T_{\min}}^{\text{exp}} \sim HN(0,10^2)$$
$$\mu_{T_{\max}} \sim N(38,10^2)$$
$$\sigma_{T_{\max}} \sim HN(0,10^2)$$
$$\sigma_{T_{\max}}^{\text{exp}} \sim HN(0,10^2)$$

For p_E we used informative hyperpriors for the between-species and between-experiment standard deviations of $\chi, T_{\min}, T_{\max}$ that draw on the estimates derived for juvenile survival (for details and reasoning we refer to our previous work [1]):

$$\mu_{\chi} \sim N(0.5,2)$$
$$\sigma_{\chi} \sim \Gamma(1.38,6.13)$$
$$\sigma_{\chi}^{\exp} \sim \Gamma(34.59,46.78)$$
$$\mu_{T_{\min}} \sim N(10,10^2)$$
$$\sigma_{T_{\min}} \sim \Gamma(10.67,2.55)$$
$$\sigma_{T_{\min}}^{\exp} \sim \Gamma(2.13,1.67)$$
$$\mu_{T_{\max}} \sim N(38,10^2)$$
$$\sigma_{T_{\max}} \sim \Gamma(7.75,2.2)$$
$$\sigma_{T_{\max}}^{\exp} \sim \Gamma(20.25,8.58)$$

The new parameter estimates per mosquito species for p_E and p_J are given in Tables S4-S5. Plots of the updated mean temperature response are provided in Figures S5-S6. The updated and previous estimates for temperature limits and the optimal temperature of R_0^{rel} for six *Culex* species are presented in Table S6. The changes to our methodology described here have only marginal impacts on the results described in our previous work and leave their general implications unchanged [1].

Table S2. Posterior mean and 95% CI of parameter estimates of hierarchical priors and of the expected temperature response by species for the juvenile survival probability $p_J(T)$ updating our estimates from previous work [1].

	$1000 * q$	T_{max}	T_{min}
Ae. nigromaculis (n_{exp}=1, n_{total}=6)	4.87 (2.66 – 8.04)	38.74 (34.04 – 45.06)	11.38 (7.60 – 14.85)
Ae. solicitans (n_{exp}=1, n_{total}=8)	4.42 (2.64 – 6.82)	39.25 (34.78 – 44.59)	10.84 (7.40 – 13.95)
Ae. triseriatus (n_{exp}=2, n_{total}=10)	4.09 (2.52 – 6.16)	35.26 (31.89 – 38.69)	5.36 (-0.17 – 9.38)
Ae. vexans (n_{exp}=1, n_{total}=6)	4.70 (2.54 – 7.78)	38.74 (33.90 – 45.07)	10.82 (7.06 – 14.39)
Cs. inornata (n_{exp}=3, n_{total}=17)	6.65 (3.78 – 10.60)	30.91 (27.50 – 34.99)	7.85 (4.70 – 10.60)
Cs. melanura (n_{exp}=1, n_{total}=7)	14.47 (5.61 – 36.43)	33.52 (29.08 – 37.72)	16.72 (11.43 – 20.99)
Cx. pipiens molestus (n_{exp}=4, n_{total}=17)	5.86 (3.54 – 8.63)	35.47 (32.61 – 38.65)	11.24 (7.93 – 13.62)
Cx. pipiens pallens (n_{exp}=2, n_{total}=10)	3.90 (2.61 – 5.66)	39.99 (36.00 – 44.25)	9.78 (6.85 – 12.49)
Cx. pipiens (n_{exp}=13, n_{total}=65)	4.04 (3.27 – 4.94)	36.87 (35.25 – 38.61)	7.08 (5.24 – 8.79)
Cx. quinquefasciatus (n_{exp}=16, n_{total}=69)	4.07 (3.26 – 5.01)	38.06 (36.41 – 39.84)	8.67 (7.01 – 10.32)
Cx. restuans (n_{exp}=5, n_{total}=25)	3.32 (2.21 – 4.69)	35.69 (33.13 – 38.50)	2.51 (-2.71 – 6.49)
Cx. salinarius (n_{exp}=1, n_{total}=8)	5.26 (3.08 – 8.68)	34.08 (29.83 – 38.20)	8.03 (3.71 – 11.68)
Cx. tarsalis (n_{exp}=3, n_{total}=9)	5.49 (3.05 – 8.73)	36.44 (32.7 – 40.93)	10.90 (6.75 – 13.92)
Population-level mean μ	1.14* (0.81 – 1.49)	36.39 (34.10 – 38.91)	9.34 (6.69 – 11.93)
Between-species variability σ	0.22* (0.01 – 0.67)	3.53 (1.49 – 6.40)	4.19 (2.14 – 7.24)
Between-experiment variability σ^{exp}	0.74* (0.53 – 1.03)	2.36 (1.44 – 3.54)	1.28 (0.09 – 2.79)

*hierarchical priors are defined for χ instead of q directly

Table S3. Posterior mean and 95% CI of parameter estimates of hierarchical priors and of the expected temperature response by species for the egg viability $p_E(T)$ updating our estimates from previous work [1].

	$\mathbf{1000 * q}$	$\boldsymbol{T}_{\mathbf{max}}$	$\boldsymbol{T}_{\mathbf{min}}$
Cx. pipiens molestus (n_{exp}=3, n_{total}=19)	4.74<br>(2.30 – 8.22)	33.82<br>(30.64 – 37.41)	5.14<br>(-3.26 – 10.54)
Cx. pipiens pallens (n_{exp}=1, n_{total}=7)	3.10<br>(1.93 – 4.72)	40.19<br>(35.69 – 45.06)	5.24<br>(-0.17 – 9.77)
Cx. quinquefasciatus (n_{exp}=2, n_{total}=11)	5.78<br>(3.13 – 9.43)	38.65<br>(34.65 – 43.01)	12.80<br>(7.75 – 16.10)
Cx. theileri (n_{exp}=1, n_{total}=13)	3.45<br>(2.30 – 5.06)	40.86<br>(36.27 – 45.40)	7.74<br>(4.07 – 11.08)
Population-level mean μ	1.51*<br>(0.76 – 2.51)	38.34<br>(34.10 – 42.75)	7.87<br>(2.64 – 12.84)
Between-species variability σ	0.21*<br>(0.01 – 0.64)	3.71<br>(1.87 – 6.25)	4.28<br>(2.31 – 6.85)
Between-experiment variability σ^{exp}	0.73*<br>(0.50 – 0.98)	2.25<br>(1.40 – 3.29)	1.33<br>(0.19 – 3.49)

*hierarchical priors are defined for χ instead of q directly

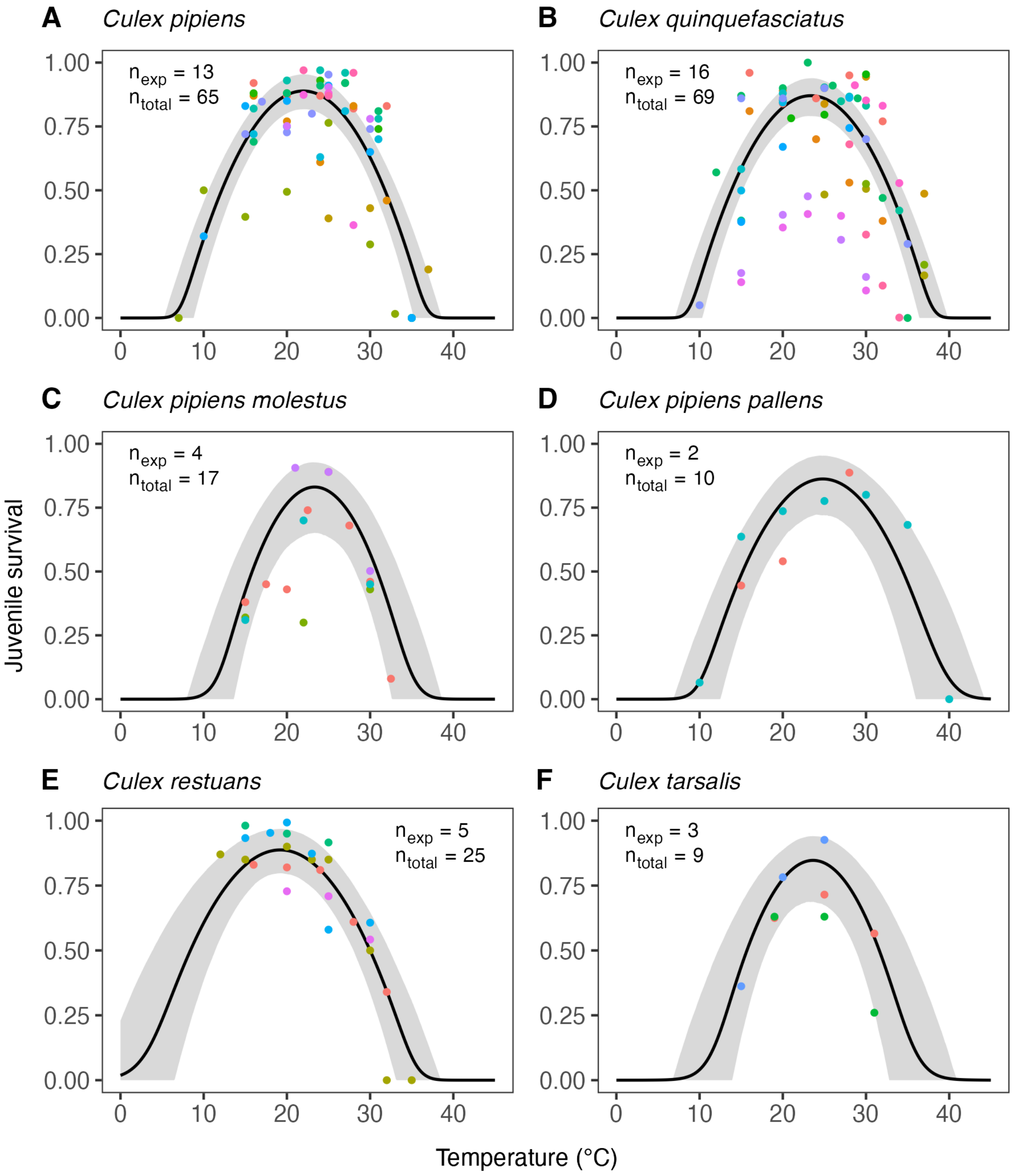

Figure S1. Estimates of the expected temperature response of larva to adult survival for six *Culex* species. Dots represent data from experimental studies, with measurements for the same species shown in different colours to indicate separate experiments. Black solid lines represent posterior distribution mean model fits. Grey shaded areas represent the corresponding central/equal-tailed 95% CI. n_{exp} and n_{total} denote the number of experiments and the total number of data points for each species.

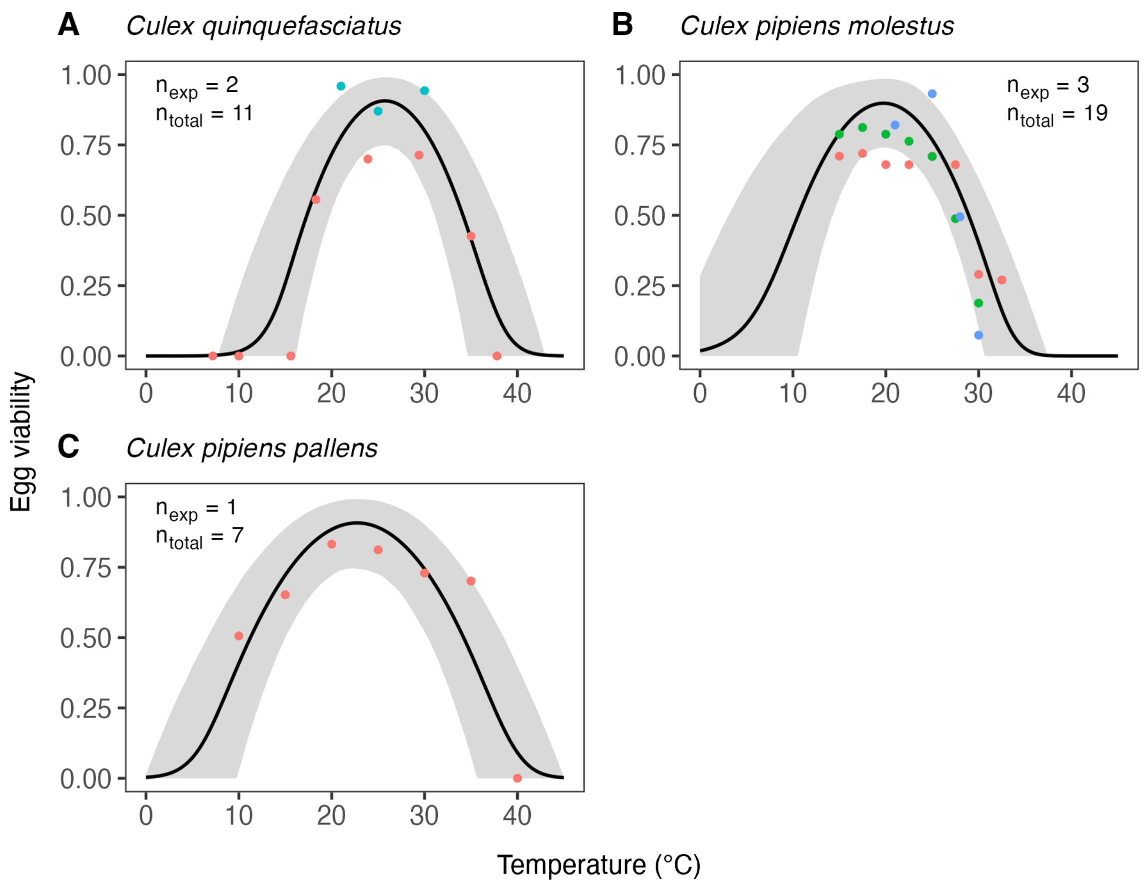

Figure S2. Estimates of the expected temperature response of egg viability for three *Culex* species. Dots represent data from experimental studies, with measurements for the same species shown in different colours to indicate separate experiments. Black solid lines represent posterior distribution mean model fits. Grey shaded areas represent the corresponding central/equal-tailed 95% CI. n_{exp} and n_{total} denote the number of experiments and the total number of data points for each species.

Table S4. Overview of updated and previous [1] estimates of the optimal temperature and temperature limits of R_0^{rel} for WNV in six *Culex* species.

Species	T^R_{min}	T^R_{opt}	T^R_{max}
Cx. pipiens (updated)	9.8 (6.3-18.1)	24.4 (22.9-25.9)	33.6 (29.9-35.5)
Cx. pipiens (previous)	9.8 (6.6-16.0)	24.5 (23.0-25.9)	33.6 (30.3-35.4)
Cx. quinquefasciatus (updated)	12.9 (9.1-16.1)	24.6 (23.4-25.8)	33.8 (31.8-35.7)
Cx. quinquefasciatus (previous)	12.5 (9.3-15.6)	24.5 (23.2-25.8)	33.8 (31.8-35.6)
Cx. pipiens molestus (updated)	11.3 (8.5-13.7)	23.6 (21.8-25.3)	32.2 (29.5-34.4)
Cx. pipiens molestus (previous)	10.7 (8.5-12.9)	23.7 (21.9-25.6)	32.1 (29.6-34.1)
Cx. pipiens pallens (updated)	10.0 (7.6-12.6)	24.7 (22.9-26.8)	34.4 (31.3-37.7)
Cx. pipiens pallens (previous)	10.2 (8.0-12.7)	24.9 (22.9-27.2)	34.4 (31.3-37.5)
Cx. restuans (updated)	9.0 (3.3-18.1)	23.5 (20.4-26.1)	31.9 (26.8-35.4)
Cx. restuans (previous)	9.4 (4.9-16.1)	23.5 (20.4-26.1)	31.9 (26.8-35.1)
Cx. tarsalis (updated)	12.0 (8.1-18.1)	25.5 (23.3-27.7)	34.4 (29.3-37.6)
Cx. tarsalis (previous)	11.3 (8.4-16.0)	25.6 (23.4-27.8)	34.5 (29.6-37.3)

C Additional country-level and sensitivity results

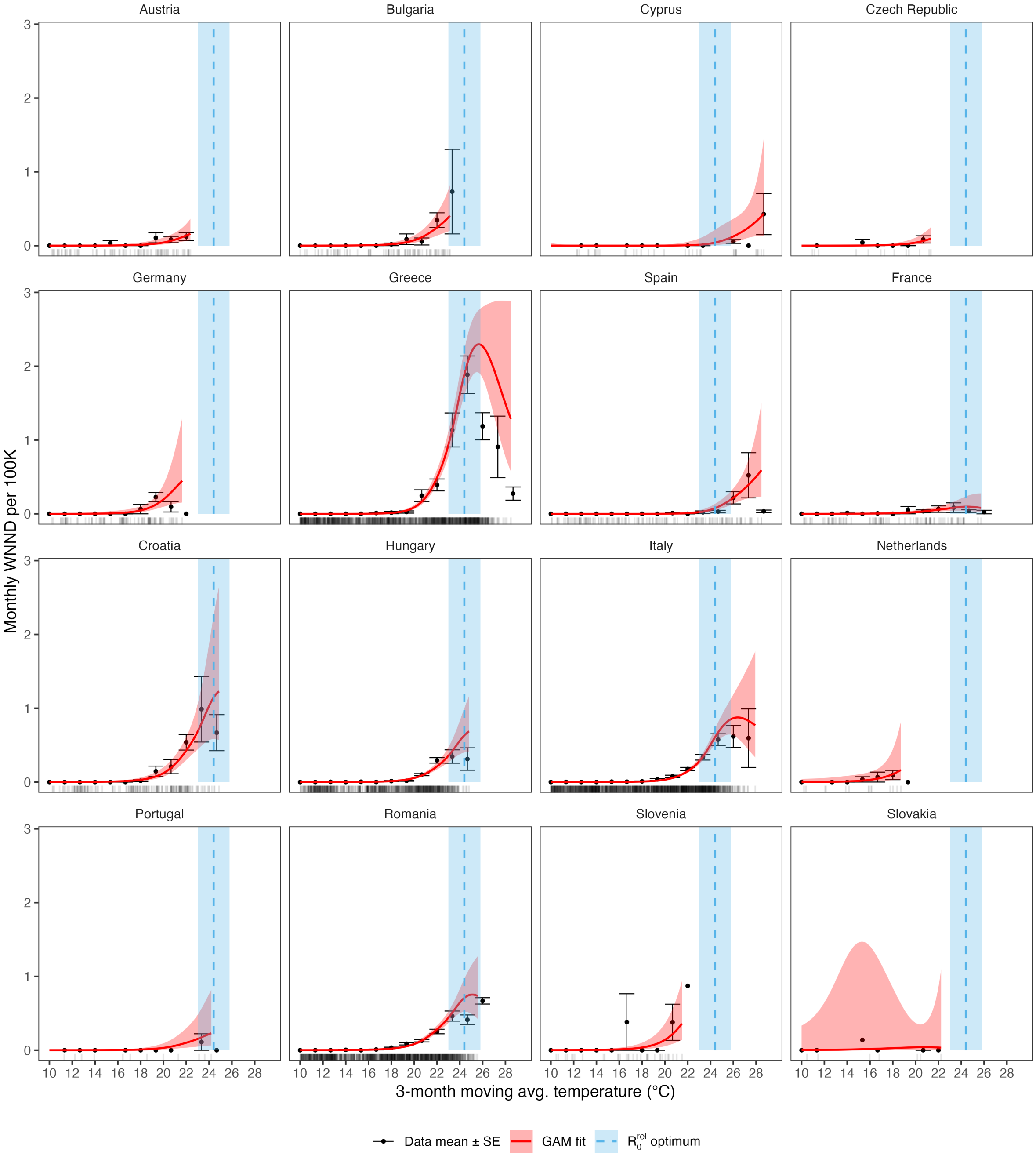

Figure S3. The response of the monthly WNND incidence to 3-month moving average temperature by country with adjustment to additional variables. Binned data means ± standard errors for equally distant bins (black dots and bars), GAM mean predictions with approximate 95% CI (red solid lines and ribbons) and mean and 95% CI of the optimal temperature of R_0^{rel} (blue dashed line and ribbon). The rug plots on top of the x-axes show the distribution of 3-month moving average temperature.

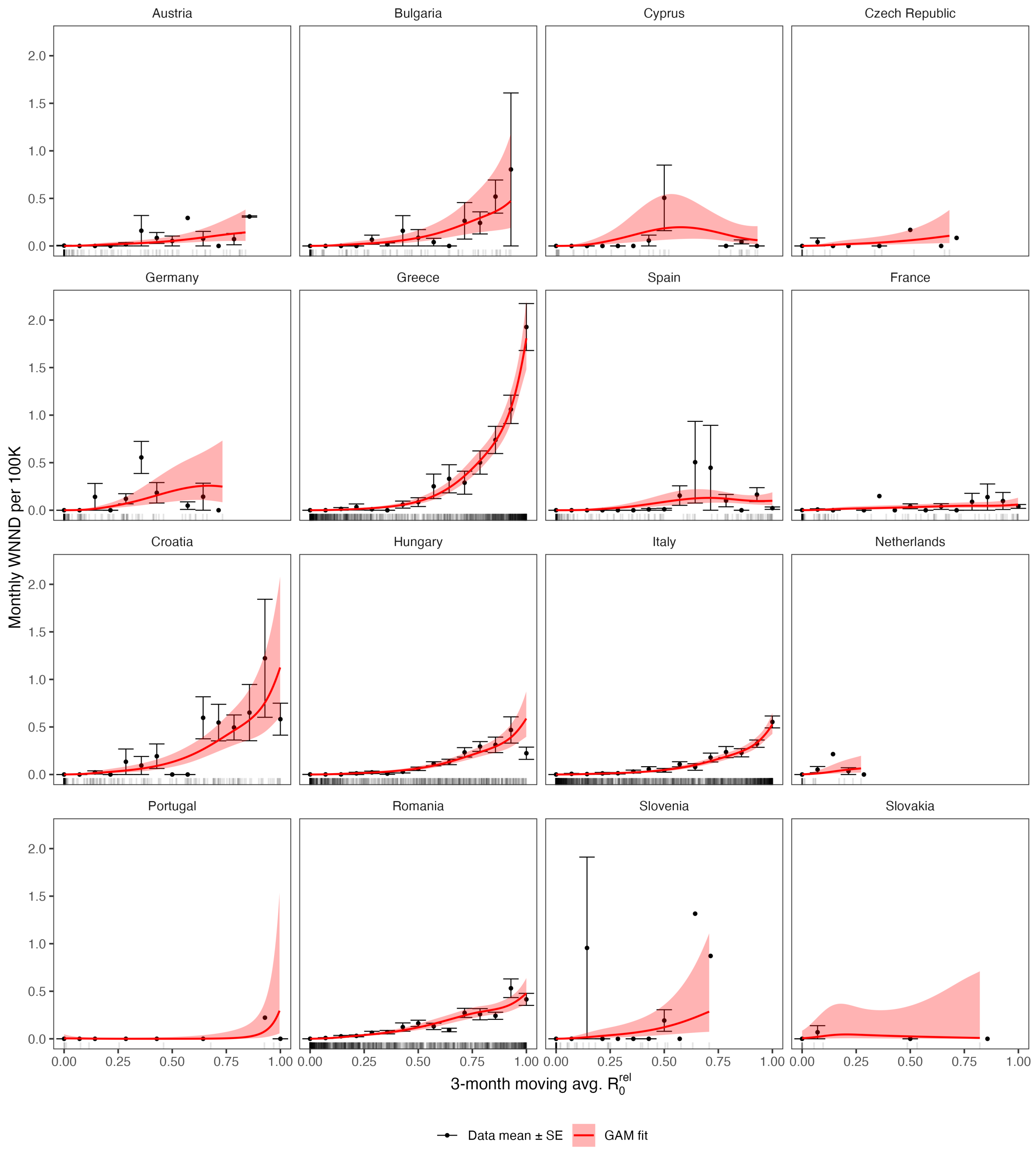

Figure S4. The response of the monthly WNND incidence to 3-month moving average R_0^{rel} by country. Binned data means ± standard errors for equally distant bins (black dots and bars) and GAM mean predictions with approximate 95% CI (red solid lines and ribbons). The rug plots on top of the x-axes show the distribution of 3-month moving average R_0^{rel}.

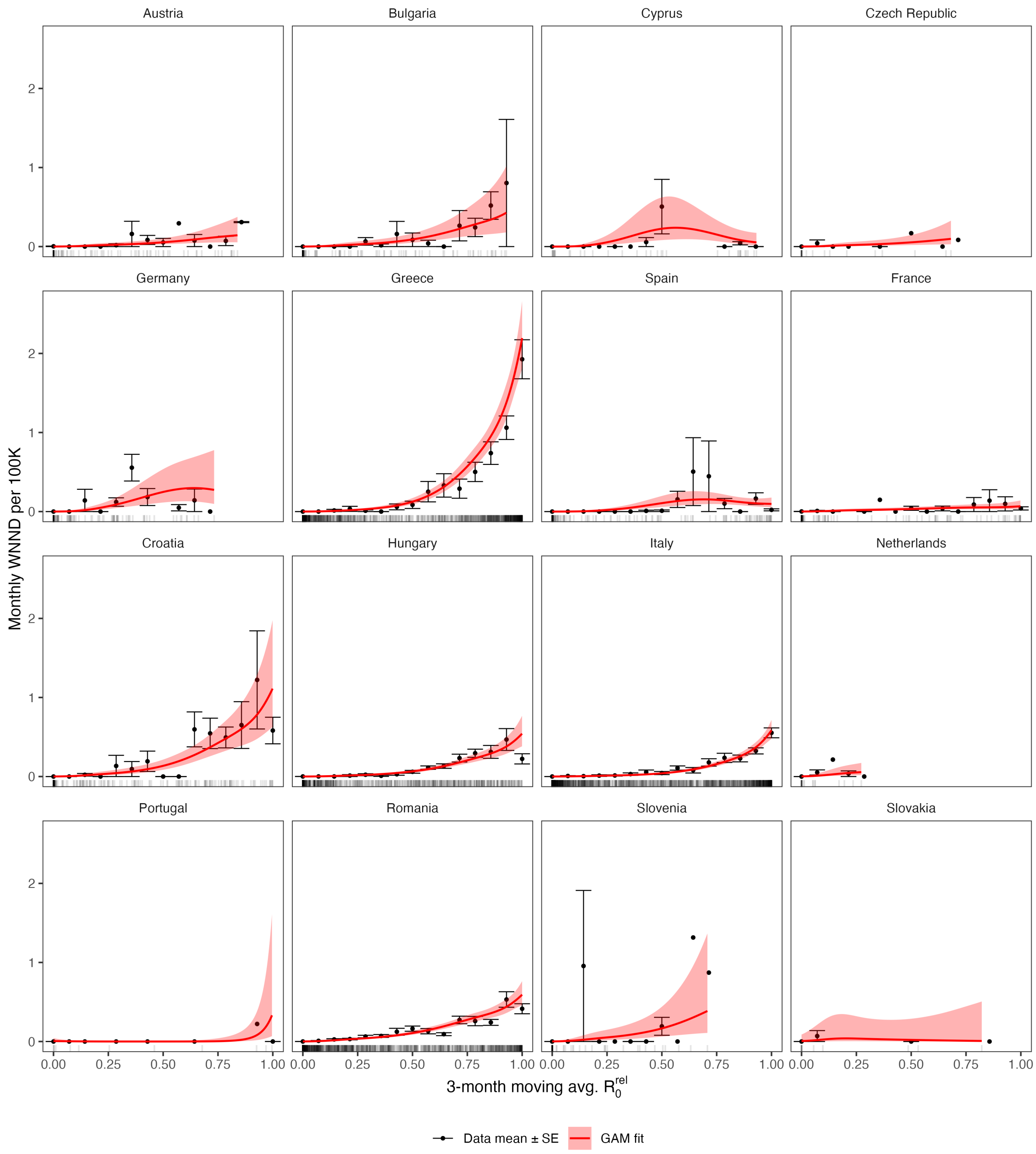

Figure S5. The response of the monthly WNND incidence to 3-month moving average R_0^{rel} by country with adjustment to additional variables. Binned data means ± standard errors for equally distant bins (black dots and bars) and GAM mean predictions with approximate 95% CI (red solid lines and ribbons). The rug plots on top of the x-axes show the distribution of 3-month moving average R_0^{rel}.

Table S5. Rank correlation coefficients of 3-month moving average temperature and R_0^{rel} with the monthly WNND incidence after excluding observations from Greece. Correlation coefficients within the same column that share the same superscript letter are not significantly ($p > 0.05$) different from one another.

**Predictor**	**Kendall's τ_B with monthly WNND incidence**	
	No restriction	Limited to temperatures >15°C
Temperature	0.407^{a}	0.387^{abc}
$R_{0,\ \mathrm{nuts3}}^{\mathrm{rel,\ daily}}$	0.411^{e}	0.404^{e}
$R_{0,\ \mathrm{nuts3}}^{\mathrm{rel,\ monthly}}$	0.417^{f}	0.393^{c}
$R_{0,\ \mathrm{nuts3}}^{\mathrm{rel,\ window}}$	$\mathbf{0.434^{g}}$	0.385^{b}
$R_{0,\ \mathrm{grid}}^{\mathrm{rel,\ daily}}$	0.411^{b}	$\mathbf{0.406^{d}}$
$R_{0,\ \mathrm{grid}}^{\mathrm{rel,\ monthly}}$	0.415^{c}	0.395^{a}
$R_{0,\ \mathrm{grid}}^{\mathrm{rel,\ window}}$	0.429^{d}	0.386^{b}

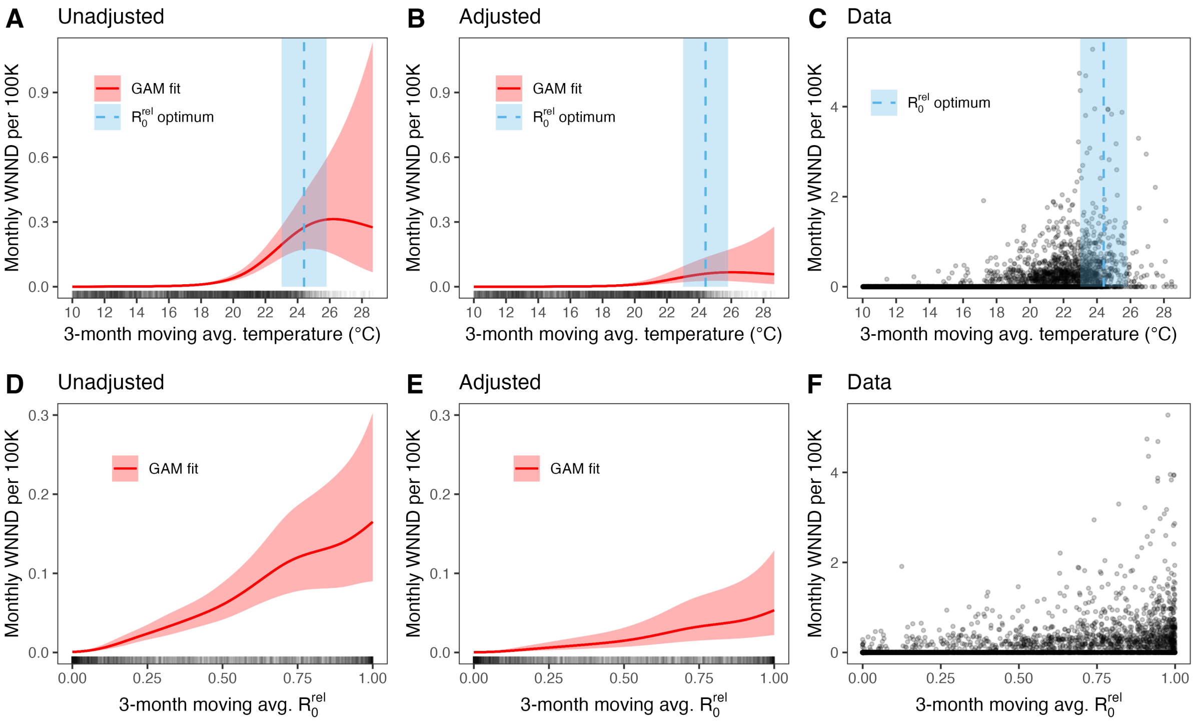

Figure S6. The shared response of the monthly WNND incidence to 3-month moving average temperature (A, B) and R_0^{rel} (D, E) without (A, D) and with (B, E) adjustment to additional variables after excluding observations from Greece. GAM mean predictions with approximate 95% CI (red solid line and ribbon) and mean and 95% CI of the optimal temperature of R_0^{rel} (blue dashed line and ribbon). Rug plots on top of x-axes show the distribution of 3-month moving average temperature/R_0^{rel}. (C), (F) Corresponding raw data.

References

[1] J. Heidecke, J. Wallin, P. Fransson, P. Singh, H. Sjödin, P.C. Stiles, M. Treskova, J. Rocklöv, Uncovering temperature sensitivity of West Nile virus transmission: Novel computational approaches to mosquito-pathogen trait responses, PLoS Comput Biol 21 (2025) e1012866. https://doi.org/10.1371/journal.pcbi.1012866.
